# A human gut phage catalog correlates the gut phageome with type 2 diabetes

**DOI:** 10.1186/s40168-018-0410-y

**Published:** 2018-02-01

**Authors:** Yingfei Ma, Xiaoyan You, Guoqin Mai, Taku Tokuyasu, Chenli Liu

**Affiliations:** Institute of Synthetic Biology, Shenzhen Institutes of Advanced Technology, Chinese Academy of Sciences, 1068 Xueyuan Avenue, University Town, Nanshan, Shenzhen, 518055 China

**Keywords:** Phageome, Metagenomics, T2D, Alterations, Correlation

## Abstract

**Background:**

Substantial efforts have been made to link the gut bacterial community to many complex human diseases. Nevertheless, the gut phages are often neglected.

**Results:**

In this study, we used multiple bioinformatic methods to catalog gut phages from whole-community metagenomic sequencing data of fecal samples collected from both type II diabetes (T2D) patients (*n* = 71) and normal Chinese adults (*n* = 74). The definition of phage operational taxonomic units (pOTUs) and identification of large phage scaffolds (*n* = 2567, ≥ 10 k) revealed a comprehensive human gut phageome with a substantial number of novel sequences encoding genes that were unrelated to those in known phages. Interestingly, we observed a significant increase in the number of gut phages in the T2D group and, in particular, identified 7 pOTUs specific to T2D. This finding was further validated in an independent dataset of 116 T2D and 109 control samples. Co-occurrence/exclusion analysis of the bacterial genera and pOTUs identified a complex core interaction between bacteria and phages in the human gut ecosystem, suggesting that the significant alterations of the gut phageome cannot be explained simply by co-variation with the altered bacterial hosts.

**Conclusions:**

Alterations in the gut bacterial community have been linked to the chronic disease T2D, but the role of gut phages therein is not well understood. This is the first study to identify a T2D-specific gut phageome, indicating the existence of other mechanisms that might govern the gut phageome in T2D patients. These findings suggest the importance of the phageome in T2D risk, which warrants further investigation.

**Electronic supplementary material:**

The online version of this article (10.1186/s40168-018-0410-y) contains supplementary material, which is available to authorized users.

## Background

The gut microbiota has increasingly been recognized as a key contributor to human health, and various chronic human diseases can be linked to dysbiosis of the intestinal microbiota [1–7]. The human gut virome, also known as the phageome, refers to the whole community of viruses in the gut, most of which are bacteriophages. Bacteriophages are thought to be the most abundant biological entities in the human gut ecosystem. There may be at least 10^9^ virus-like particles (VLPs) per gram of human feces, ten times more than that of bacterial cells [8, 9]. However, phageome studies remain challenging because of the extremely high diversity of the phage community, highly divergent phage genomes, and lack of a universal marker akin to the bacterial 16S rRNA genes.

Recent advances in next-generation sequencing (NGS) technology and bioinformatic tools have fostered the development of large-scale studies of the human gut phageome [7, 9]. One approach based on metagenomic analysis of VLPs focuses primarily on the free phages at the time of sampling [10–14]. In whole-community metagenomic sequencing (WCMS)-based approaches, it is estimated that up to 17% of the WCMS-identified DNA sequences from stool samples are of phage origin [10, 15]. WCMS data that contain valuable information about phages, prophages, and their bacterial hosts can be used to predict the interactions between gut phages and their bacterial hosts, allowing in-depth study of the dynamics of gut microbial communities (for example, see refs. [7, 9, 16–18]).

Bacteriophages play roles in intestinal physiology that are far more important than the alteration of bacterial communities by phage infection [19–25]. For instance, phages residing in mucosal surfaces can provide non-host-derived immunity against bacterial infections [26]. A very recent study uncovered inflammatory bowel disease (IBD)-specific alterations in the enteric virome [14, 27]. Therefore, the gut phageome may play important roles in T2D, obesity, and related diseases.

Qin et al. found that T2D is linked to gut bacterial dysbiosis using a metagenomic analysis of WCMS data [3] but neglected the role of gut phages in T2D. In this study, utilizing a WCMS dataset developed from fecal samples from a total of 370 Chinese T2D patients and non-diabetic controls [3], we used three bioinformatic strategies to identify large scaffolds of phage origin and defined phage operational taxonomic units (pOTUs) to obtain insights into the human gut phageome. This is the first study to correlate the gut phageome with T2D, and further investigations are required to determine if the gut phageome contributes to T2D risk.

## Methods

### Metagenomic datasets used in this study

All WCMS reads used in this study were obtained from the NCBI short read archive (SRA) database (SRA045646) generated from a previously published Chinese T2D microbiome study [3]. The study was a case–control study that included 187 cases and 183 controls. Similar to the published study, we divided our study into two stages. Stage I was an exploration stage (control *n* = 74, T2D *n* = 71), and stage II was a validation stage (control *n* = 109, T2D *n* = 116). Additionally, the corresponding assembled scaffolds generated from the exploration stage were downloaded from GigaDB (gigadb.org/dataset/100036) [3].

The VLP-based metagenomic sequencing datasets generated in three previous studies were downloaded from the EMBL-EBI (PRJEB7772) and NCBI SRA (SRP021107, SRX020505, and SRX020504) [10, 12, 14]. These data of phage origin were used to capture new phages in the WCMS data and to compare the differences in phage distribution between the WCMS data and VLP-based sequencing data.

All datasets used in this study are listed in Additional file 1: Table S1.

### Detection of known phage genomes in the metagenome

Known bacterial phage genomes were downloaded from the European Nucleotide Archive database (ENA, http://www.ebi.ac.uk/genomes/phage.html) and formatted as the ENA phage database (ENADB), and the corresponding protein sequences were formatted as the European Nucleotide Archive Protein Database (ENAPDB). Overall, the ENADB contains 2501 phage genomes associated with 2010 bacterial host species. The WCMS reads (145 samples in stage I of the T2D study) and VLP reads (including all three VLP metagenomic datasets (Additional file 1: Table S1)) were mapped to the genomes in the ENADB by the software Soap2. The breadth of coverage and depth of coverage of each phage genome by the reads in each sample were estimated with default flags [3, 10, 12, 14, 28]. Only the ENA genomes with > 30% breadth of coverage were selected for display.

### Identification of large phage scaffolds (≥ 10 kb)

**Fig. 1:**
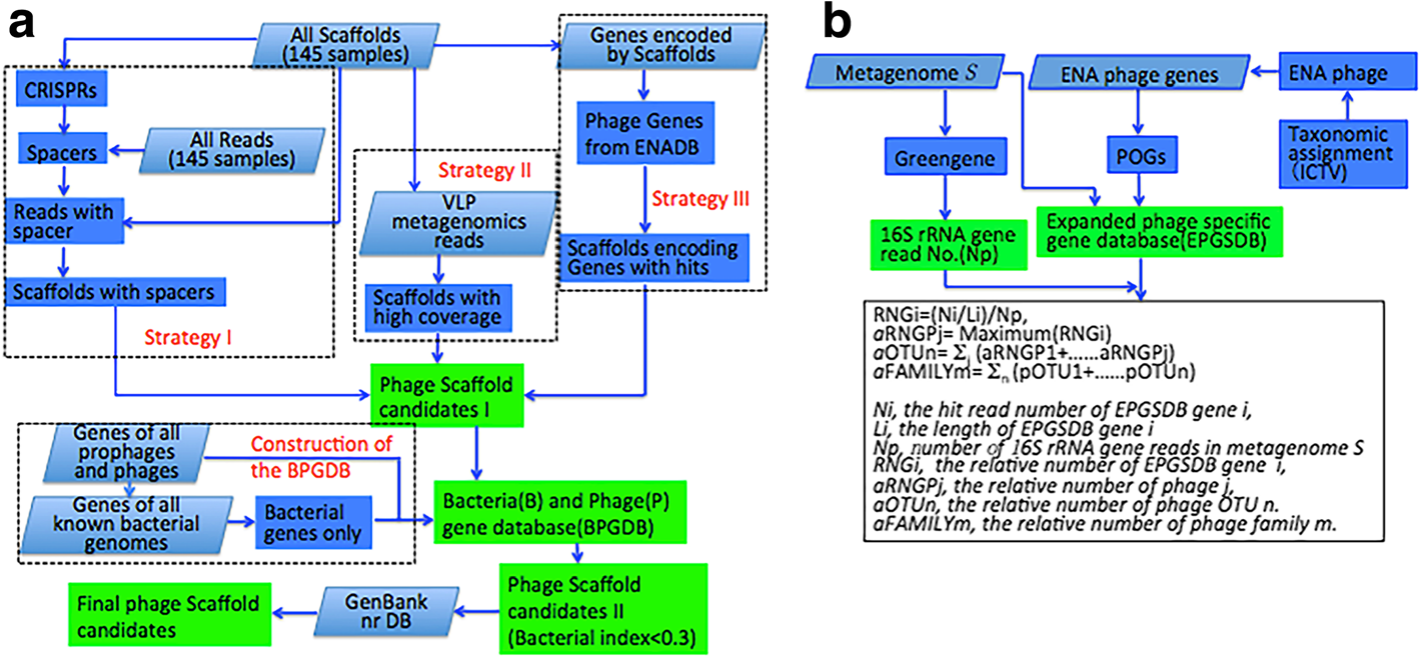
Identification of the large phage scaffolds and definition of pOTU. **a** The pipeline used three strategies to identify the large phage scaffolds (≥ 10 k) (see also supplementary Materials and Methods for details). In strategy I, the scaffolds were probed by CRISPR spacer(s). In strategy II, the scaffolds were mapped by reads from the VLP metagenome with a high breadth of coverage (≥ 40%). In strategy III, the scaffolds encoding genes homologous to those of ENA phages were identified. The Bacteria and Phage Gene Database (BPGDB) was constructed. The assigned scaffolds were manually checked by comparing them against the BPGDB and GenBank nr database. The scaffolds with ambiguous assignments were discarded in the next analysis steps. **b** Definition of pOTUs to profile the phageome on the read level (see also supplementary Materials and Methods for details). The Expanded Phage-Specific Gene database (EPSGDB) was constructed according to the Phage Orthologous Groups (POGs). The phage taxonomical classification was performed based on the International Committee on Taxonomy of Viruses (ICTV) [17, 30]. The relative number of a pOTU in a sample was calculated by summing the numbers of all phage genomes belonging to the pOTU and dividing by the number of 16S rRNA gene reads in the sample

Three strategies were developed to identify large scaffolds (≥ 10 kb) of putative phage origin in the assembled scaffolds of the T2D study (gigadb.org/dataset/100036) (Fig. 1a; see also Additional file 1: Materials and Methods for details). Briefly, we identified a large scaffold (≥ 10 kb) as a candidate sequence of phage origin if (1) the scaffold was probed by spacer(s) of CRISPRs, (2) the scaffold was mapped by VLP metagenomic reads with a high breadth of coverage (≥ 40%), and (3) the scaffold encoded gene(s) homologous to the gene(s) from ENA phages. Finally, all assigned scaffolds were manually checked, and the scaffolds with ambiguous assignments were discarded in the next analysis steps. Because most of the identified phage scaffolds in this study are part of phage genomes, we did not intend to distinguish between phage and prophage sequences. This process identified 2567 putative large phage scaffolds from the 145 stage I samples.

The relative abundance of each identified phage scaffold was estimated. To this end, we firstly mapped all reads to the identified phage scaffolds and counted the numbers of the reads mapping to the identified phage scaffolds in each sample, respectively; secondly, the read number corresponding to each identified phage scaffold was normalized by the total read number of each sample; the normalized value thereby represents the relative abundance of each phage scaffold in each sample.

### Definition of phage operational taxonomic units and profile of bacterial genera

**Fig. 2:**
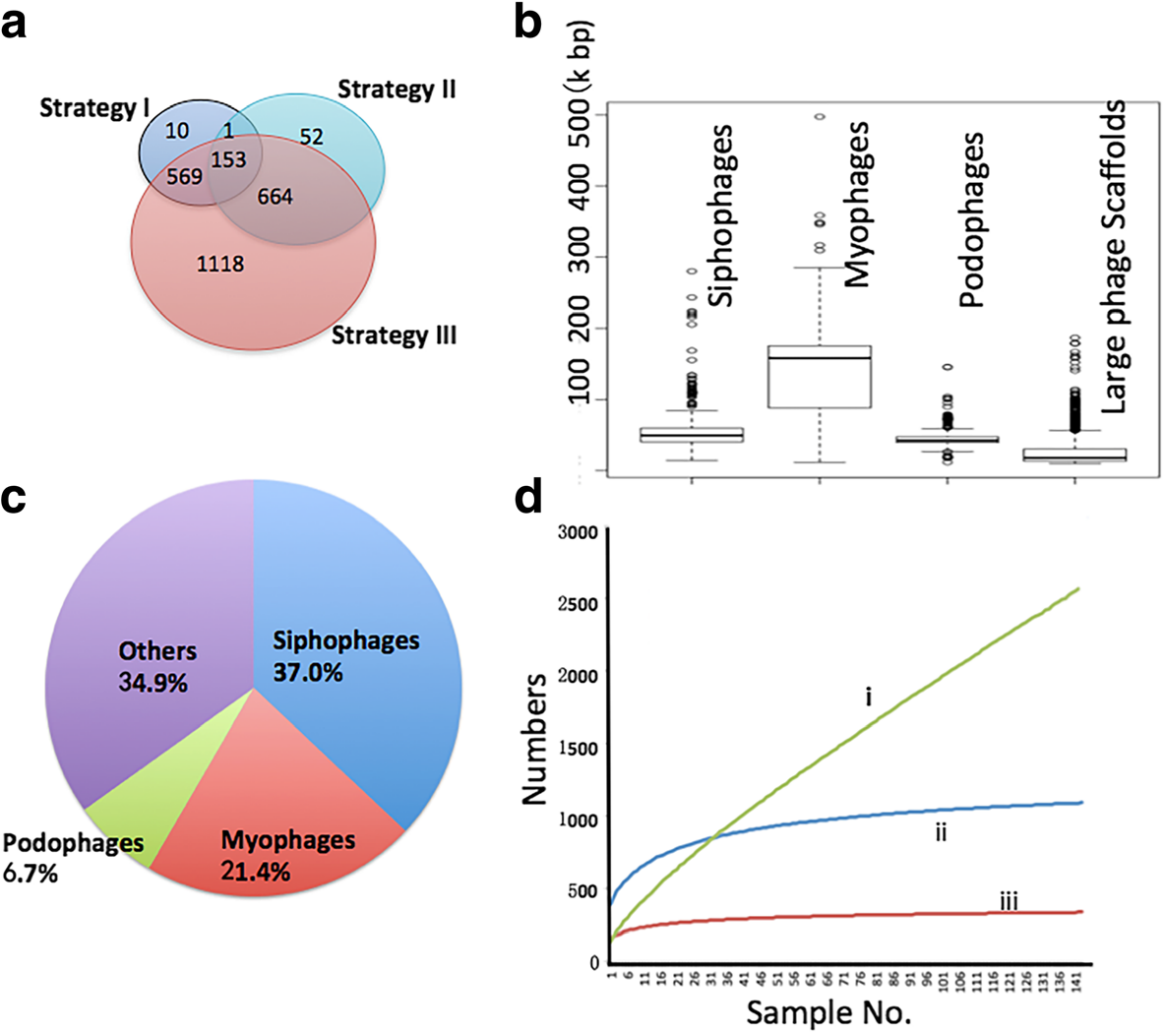
Statistics of the identified phageome in the samples. **a** Venn diagram showing the numbers of the large phage scaffolds identified by the three strategies. **b** Length range of the genomes of siphophages, myophages, podophages, and the identified large phage scaffolds. **c** The percentage of phage families identified by the large phage scaffolds. **d** Rarefaction curves of the phageome, based on the number of the identified large phage scaffolds (i), the number of ENA genomes with a high breadth of coverage in the metagenomes (ii), and the number of defined pOTUs in the metagenomes (iii)

An operational taxonomic unit (OTU) is defined as a group of closely related individuals that share a given set of observed characters or undetermined evolutionary relationships [29]. We bioinformatically defined phage OTUs (pOTUs) (Fig. 2b; see also Additional file 1: Materials and Methods for details) to profile the phageome and examine the interactions between phages and bacteria in the gut ecosystem. Briefly, we constructed an Expanded Phage-Specific Gene database (EPSGDB) according to the Phage Orthologous Groups (POGs) and the phage classification defined by the International Committee on Taxonomy of Viruses (ICTV) [17, 30]. A pOTU was defined as the collection of all phages with the same taxonomic names at all five levels (group|order|family|subfamily|genus) and sharing hosts within the same genera and a pOTU is not necessary to be associated with species or genus. Thus, a pOTU was named with an ordered list of phage names at all the five levels plus the genus name of the bacterial host. In brief, we defined a pOTU to represent a group of phages with homologous phage taxon-specific genes and the same bacterial genus of their hosts. The relative abundance of a pOTU in a sample was calculated by summing the numbers of all phage genomes belonging to the pOTU and dividing by the number of 16S rRNA gene reads in the sample.

The MetaPhlAn software was used to profile the bacterial genera for each sample based on metagenomic reads with default flags [31].

### Gene annotation of the putative phage scaffolds

The genes on the putative large phage scaffolds were predicted by MetaGeneMark with default flags [32], followed by functional annotation performed by comparing the genes to various databases, including the GenBank nr database, COG (Clusters of Orthologous Groups) database [33], Tigr (The Institute for Genomic Research) Microbial Database [34], and Pfam database of conserved amino acid motifs [35], using RPSblast (Reversed Position Specific Blast) with an *e*-value cutoff of 1e−10 [36]. Virulence genes were identified by comparing the genes against the VFDB (Virulence Factor Database) with an *e*-value cutoff of 1e−05 [37].

### Taxonomic assignment of the large phage scaffolds and the bacterial scaffolds (≥ 10 kb)

We taxonomically assigned each putative large phage scaffold (≥ 10 kb) on the phage family level. All the genes encoded by the phage scaffolds were queried against the EPSPDB with an *e*-value cutoff of 1e−05. Only the genes encoded by phage scaffolds with hits in the EPSPDB and only the top two hits were taken into consideration for the taxonomic assignment. The phage family of each hit was determined using the taxonomic information provided by the EPSPDB. Each gene encoded by the phage scaffolds contributed to the scaffold taxonomic assignment based on its hits, with the top hit weighted as 1 and the second as 0.5. The phage family ratio (PFR) of a phage scaffold was calculated by normalizing the summed scores of each phage family by the total scores of the scaffold. A scaffold was assigned to a phage family if the PFR was larger than 50%. All scaffolds with PFRs of no more than 50% were assigned as unclassified.

The taxonomy of the bacterial large scaffolds (≥ 10 kb) was assigned on the genus level using an approach similar to that of the phage scaffold assignment with modifications. All the genes encoded by the bacterial scaffolds were queried against the NCBI nr database, and the bacterial genus ratio (BGR) was calculated by normalizing the summed scores of each bacterial genus by the total scores of each bacterial scaffold, with a BGR ≥ 70% as the threshold for genus assignment.

### Construction of phylogenetic trees of gut phages based on large subunit terminase sequences

Large subunit terminase (LST) sequences were selected as the marker to build phylogenetic trees of gut phages. The protein sequences annotated as LST were extracted from all ENA phage sequences. Pfam domains were searched using the hmmsearch program in the HMMER3 package (*e*-value cutoff 1e−05), and four domains, including Terminase_1 (PF03354), Terminase_3 (PF04466), Terminase_6 (PF03237) and Terminase_GpA (PF05876), were found on the LST sequences [38]. Thereafter, all the protein sequences encoded by the large phage scaffolds were searched against the Pfam database, and the protein sequences with one of the four functional domains were included in the tree construction. The alignment was performed and maximum likelihood trees were constructed by the program MEGA with 1000 bootstraps [39]. The trees were visualized by the Figtree software (http://tree.bio.ed.ac.uk).

### Construction of the interaction network of the gut bacteria and phages

The co-correlation/exclusion between the gut bacteria and phages were calculated based on the relative numbers of bOTUs and pOTUs by SparCC (with *p* < 0.01 and correlation > 0.3) [40]. Only the bacterial genera and pOTUs with a high frequency (detected in more than 72 samples (≥ 50%)) were considered. The network layout was calculated and visualized using a circular layout by the Cytoscape software [41]. Only edges with correlations greater than 0.3 and a *p* value less than 0.01 were shown, and unconnected nodes were omitted.

### Statistical analyses and data visualization

The richness of the phageome in the 145 samples of stage I was estimated by the R package Vegan based on the Chao2 richness estimator. Two rarefaction curves of the gut phageome in the control samples and T2D samples were generated. The linear discriminant analysis (LDA) scores of the variations of bacterial genera and pOTUs between control samples and T2D samples were calculated and visualized by LEfSe (LDA Effect Size) [42]. Based on the relative abundances of the highly prevalent pOTUs detected in more than 120 samples (70% of 145 samples), the significance of the variations of the gut phages between T2D and control groups were assessed by the Mann–Whitney rank-sum test with FDR correction [43]. The significant variation of the relative abundances of the identified phage scaffolds between the T2D and control groups was assessed by the Mann–Whitney rank-sum test.

## Results

### The human gut harbors a complex phageome

First, we estimated the gut phageome by mapping the WCMS reads against the known phage genomes in the European Nucleotide Archives genome database (ENADB). In the 145 WCMS metagenomes used for the stage I analysis, a total of 461 phage genomes were mapped with at least one read, with an average of 67 (± 26) per sample. A total of 438 phage genomes in the ENAPDB were mapped by at least one read of the VLP metagenomic data [10, 12, 14]. As shown in the figure (Additional file 2: Figure S1), most double-stranded (ds) DNA phage genomes exhibited a low breadth of coverage (< 20%) in both the VLP and WCMS data. Only a small fraction of the metagenomic reads (0.55% reads of the WCMS data and 0.12% of the VLP data) could be mapped to the ENADB genomes. Considering that up to 17% of sequences in the WCMS data and most of the VLP metagenomic reads may be of phage origin [10, 15], these results suggest that most of the phages in the human gut are novel and are not yet included in the European Nucleotide Archives protein database (ENAPDB).

We next defined pOTUs to profile the gut phageome (Fig. 1b). In total, in the 145 WCMS samples, 341 pOTUs were assigned, averaging 96.7 (± 26) pOTUs per sample. A majority of the pOTUs (87.8 ± 5.6%) belonged to the order *Caudovirales*. *Siphoviridae* was the most abundant family, accounting for 55.3 ± 9.8% of all identified phages, followed by *Myoviridae* (21.7 ± 9.9%) and *Podoviridae* (10.6 ± 8.4%) (Additional file 2: Figure S2).

Finally, using three bioinformatic strategies, we identified large phage scaffolds from the assembled scaffolds of the WCMS data for the stage I analysis (Fig. 1a). A total of 2567 scaffolds larger than 10 kb were putatively assigned as phage in origin (Fig. 2a, Additional file 3: Table S2). The large phage scaffolds ranged from 10 to 187 kb in length, which is consistent with the length range of phage genomes in the order *Caudovirales* (Fig. 2b). We taxonomically assigned each large phage scaffold as described in the “Methods” section. In total, 1671 of the 2567 (65.1%) large phage scaffolds were qualified to assign on the family level. As observed for the pOTUs, the phages of *Siphoviridae* produced the largest number of scaffolds (950, 37.0%), followed by *Myoviridae* (549, 21.4%) and *Podoviridae* (172, 6.7%) (Fig. 2c).

We estimated the richness of the identified phageome in this study. As shown in Fig. 2d, the rarefaction curve of the phageome, based on the number of the identified large phage scaffolds, grows rapidly across the 145 samples, suggesting that the gut phages are highly individualized. Based on the number of ENA genomes with a high breadth of coverage in the metagenomes and the number of defined pOTUs in the metagenomes, the rarefaction curves reach a plateau within approximately 20 samples, indicating that the pOTUs that we defined in this study are sufficient to represent the gut phageome.

### Analyses of the large phage scaffolds identify novel phagetypes and vast genetic diversity in the gut phageome

In the 145 WCMS samples, most of the large phage scaffolds identified were unique to each sample, with a few exceptions of phage scaffolds widely distributed among individuals (Additional file 2: Figure S3a and b). The phages with a high breadth of coverage and depth of coverage were considered to be active at the time of sampling or to be prophages on bacterial genomes of some abundant taxa, such as *Bacteroides* spp*.* The newly identified crAssphage was one of the most prevalent phages and was observed in 23.4% (*n* = 34) of the samples, and the largest crAssphage scaffolds were 90–100 kb in length, in line with the length of their reported prototype genome [44].

**Fig. 3:**
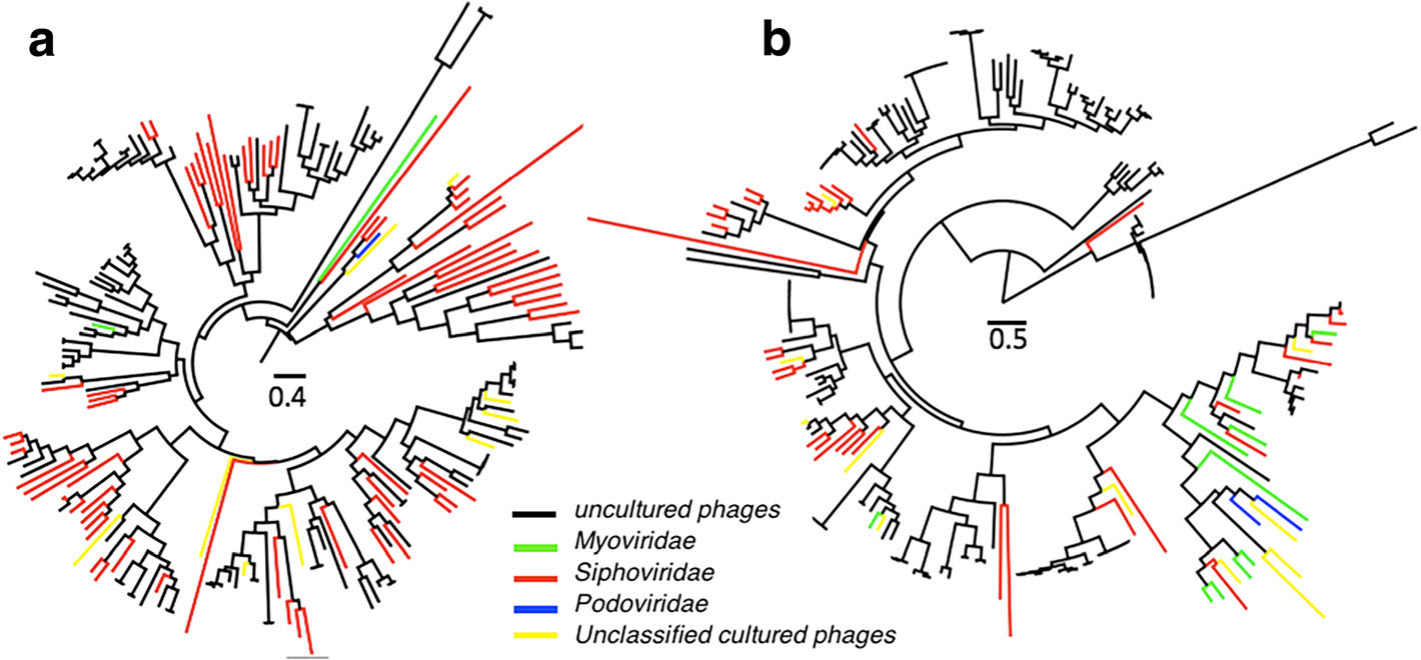
Phylogenetic trees of gut phages based on large subunit terminases. **a** Terminase_1; **b** Terminase_3; uncultured, black; *Myoviridae*, green; *Siphoviridae*, red; *Podoviridae*, blue. Unclassified cultured, yellow. Maximum-likelihood trees of all identified large subunit terminases from the identified large phage scaffolds and ENA phage genomes. The red branches in the trees denote the sequences from ENA phage genomes

To establish the phylogeny and estimate the diversity of the uncultured gut phages, we selected large subunit terminase (LST) as a marker to construct phylogenetic trees. The LST sequences identified in the ENADB phage genomes had four different Pfam functional domains, including Terminase_1, Terminase_3, Terminase_6, and Terminase_GpA. In total, 576 LSTs were identified on the 2567 large phage scaffolds, with 111 belonging to the Terminase_1 domain, 145 to Terminase_3, 275 to Terminase_6, and 45 to Terminase_GpA. Phylogenetic trees of gut phages were constructed according to these domains (Fig. 3 and Additional file 2: Figure S4). As shown on the trees, some clusters did not include any known ENADB phages, likely representing novel phagetypes. The LST phylogeny expanded the diversity of gut phages and defined new lineages. Interestingly, there was no clear link between the types of terminases and phage families, highlighting the potential genetic plasticity of gut phages. Possible horizontal gene transfer might have obscured some robust phylogenetic signals of gut phages.

The genes encoded by the phage scaffolds were annotated by querying the sequences against general databases, including Pfam, COG, TIGR, and Bactnog. In total, of the 83,957 genes predicted from the phage scaffolds, only 19.3% generated hits in the Expanded Phage-Specific Gene database (EPSDB), 18.8% in COG (Additional file 2: Figure S5), 15.4% in Tigr, 27.2% in Pfam, and 29.7% in the Bactnog database. Comparatively, 60.4% of genes in the integrated gene catalog of gut metagenomes generated hits in the Bactnog database [45], suggesting that most identified phage gene sequences are novel.

The large phage scaffolds identified in this study provided an opportunity to gain insights into the accessory gene pool carried by gut phages. A ubiquitous gene found in the large phage scaffolds encoded an IgA protease (Additional file 3: Table S3), whose putative function is to allow the bacterial hosts or phages to adhere to mucous membranes. The prevalence of this gene suggests that it may play an important role in gut microbiota and possibly serve as a marker for human gut phages. Another interesting accessory gene annotated as hemolysin (45% identity to WP_044154808.1) was observed in a large phage scaffold (SRR341696|Scaffold32710_3, 179 kb) that appeared in 16 WCMS samples. Hemolysin is suggested to cause lysis of red blood cells by destroying their cell membranes. The genes encoded by the phage scaffolds were compared against the VFDB with a cutoff of 1e−05, and 411 scaffolds had at least one hit to various virulence genes (Additional file 3: Table S3), which is suggestive of a gut pathogenic gene pool carried by phages.

### Phage–bacteria partnership indicates phage specificity to their bacterial hosts

**Fig. 4:**
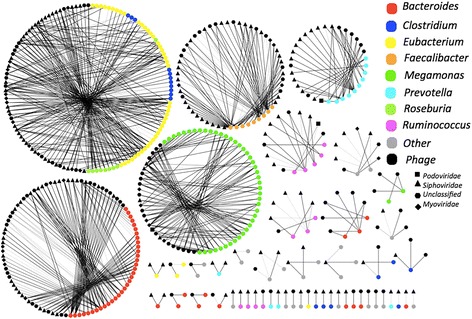
The phage–bacterium specificity networks between the identified gut phages and the inferred hosts. Only large scaffolds (≥ 10 kb) were considered. Spacers were extracted from CRISPR arrays and used to probe the corresponding phage scaffolds. Both phage and bacteria scaffolds were taxonomically assigned to the genus level. The networks were visualized by Cytoscape software. The width of the edges is proportional to the number of spacers of a bacterial scaffold paired to a phage scaffold. Only the bacterial scaffolds that could be assigned taxonomically were included

Based on the CRISPR-targeting method described by Stern et al. (Fig. 1a, Strategy I) [18], we paired the large phage scaffolds to their putative bacterial hosts (Fig. 4). In the 145 WCMS samples, we identified 1037 spacer sequences from 322 large bacterial scaffolds. These large bacterial scaffolds were generated from 112 metagenomic samples. We taxonomically assigned the large bacterial scaffolds as described in the “Methods” section, and 224 (69.6%) of them were unambiguously assigned on the genus level. The most abundant genera were *Bacteroides* (*n* = 47), *Eubacterium* (*n* = 47), *Megamonas* (*n* = 45), *Clostridium* (*n* = 18), *Prevotella* (*n* = 14), *Ruminococcus* (*n* = 12), *Faecalibacterium* (*n* = 9), and *Roseburia* (*n* = 9).

Subsequently, the obtained spacer sequences were mapped to the large phage scaffolds. Only 10 (0.96%) spacers and the paired phage scaffolds co-existed in the same WCMS samples (Fig. 4, Additional file 3: Table S4), suggesting that the spacers carried by CRISPRs provide the whole gut bacterial community highly effective protection from being infected by the corresponding phages. As shown in Fig. 4, the large phage scaffolds showed high specificity to the inferred hosts. Most (98%, 314/321) of the phage scaffolds were paired to bacterial hosts within the same genera, while 7 phage scaffolds were paired to bacterial scaffolds from two genera (*Eubacterium*–*Roseburia* (*n* = 4), *Eubacterium*–*Clostridium* (*n* = 2), *Coprococcus*–*Lachnospira* (*n* = 1)) (Fig. 4, Additional file 3: Table S4). However, these genera all belong to the order *Clostridiales*. Although wrong pairs due to short spacers cannot be excluded completely, this case implies that some gut phages are capable of infecting hosts across the genera in the order *Clostridiales*. Bacterial scaffolds of *Bacteroides* had the highest number (25%, 77/308) of the paired phage scaffolds. These *Bacteroides* phages encode many functional accessory genes, such as anaerobic ribonucleoside-triphosphate reductase, implying the importance of *Bacteroides* phages in the gut ecosystem (Additional file 2: Figure S6). In conclusion, this network analysis reveals a global pattern of gut phage specificity to their bacterial hosts in the human gut environment.

The spacers in CRISPR arrays record the history of phages infecting bacterial hosts. We observed that 65% (*n* = 275) of the 421 phage scaffolds were linked to 2–38 bacterial scaffolds and 72% (*n* = 239) of the 321 bacterial scaffolds were “attacked” by multiple phage scaffolds (*n* ≥ 2) (Fig. 4, Additional file 3: Table S4). As shown in Fig. 4, most bacterial genera can be infected by phages from all three families in the order *Caudovirales*. A number of bacterial scaffolds harbor CRISPR arrays with multiple spacers matching to the same phage scaffolds (Fig. 4, Additional file 3: Table S4). These observations demonstrated a close and co-evolutionary interaction between phages and bacteria existing in the human gut.

### Significant alterations of the gut phageome in T2D were observed and validated

**Fig. 5:**
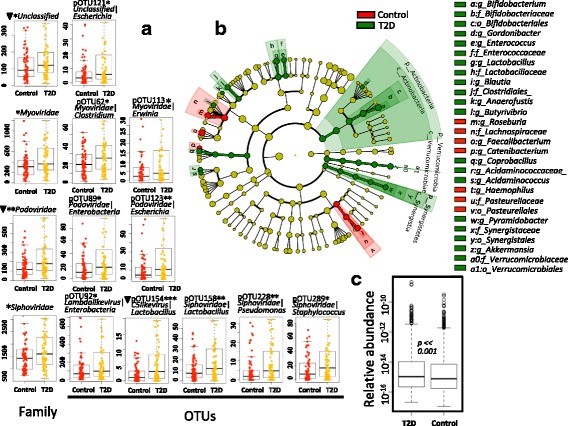
Alterations of the phage and bacterial taxa in the T2D group and control group. **a** The pOTUs whose relative numbers increased in T2D samples. *< 0.05, **< 0.01, ***< 0.001, FDR < 0.25, triangle: FDR < 0.05; Wilcoxon rank-sum test corrected by the Benjamini and Hochberg method. Only the highly prevalent pOTUs detected in 70% of the samples were included. **b** Alterations of bacterial taxa identified by LEfSe. Each circle’s diameter is proportional to the taxon’s abundance. Differences are represented in the color of the most abundant class. **c** The significant variation of the abundances of the identified phage scaffolds between the T2D group and control group; *p* value = 8.505e−11. In each WCMS sample, the phage abundance was calculated by normalizing all read numbers mapped to the identified large phage scaffolds against the total read number of the WCMS sample

A previous study indicated that the gut bacteriome is associated with T2D [3]. Considering the predator–prey relationship between the gut phages and bacteria, we expected to observe a reciprocal interaction between the phageome and bacteriome in the control and T2D samples. We first profiled the gut phageome in the human gut samples by defining pOTUs. The relative number for each pOTU in the samples was calculated according to the protocol described in the “Methods” section. Interestingly, we found that the relative numbers of the *Myoviridae*, *Podoviridae*, *Siphoviridae*, and unclassified_*Caudovirales* families increased significantly (*p* < 0.05, FDR < 0.25, Wilcoxon test) in the T2D samples (Fig. 5a). The phage alterations were further traced to the lowest-level taxon. We defined the core pOTUs (*n* = 58) as those that existed in more than 2/3 (*n* > 96) of the samples. Of these, 7 core pOTUs (4 *Siphoviridae*, 2 *Podoviridae*, and 1 unclassified family) were significantly different (*p* < 0.05, FDR < 0.25) between the T2D and control groups. Interestingly, all increased in terms of the relative number in the T2D group (Fig. 5a). The inferred bacterial hosts of these pOTUs were putatively from the bacterial genera *Enterobacteria*, *Escherichia*, *Lactobacillus*, *Pseudomonas*, and *Staphylococcus*. We reanalyzed the overall different bacterial taxa between the T2D and control samples using MetaPhlAn and LEfSe (LDA score > 3) (Fig. 5b and Additional file 2: Figure S7), showing that the alterations of the gut phageome do not agree with the changes in the putative bacterial hosts.

To validate the significant alterations, we analyzed the 7 pOTUs in a separate stage II dataset with a larger sample size, containing 225 T2D (*n* = 116) and control (*n* = 109) samples from Chinese adult individuals. We verified that all 7 pOTUs were significantly correlated (*p* < 0.05 and FDR < 0.25) with T2D (Additional file 3: Table S5). In this independent dataset, more pOTUs were associated with T2D, and the relative numbers of these pOTUs increased as well (Additional file 3: Table S5).

We also verified the significant increase in the number of phages in the T2D samples on the large phage scaffold level. The total number of reads mapped to the identified large phage scaffolds was counted and further normalized by the total read number of the samples. A Wilcoxon rank-sum test was performed to identify the difference in the normalized phage numbers between T2D and control samples. Our analysis showed a significant increase in the normalized phage number in the T2D samples (Wilcoxon test, *p* = 8.505e−11, Fig. 5c).

### Co-occurrence/exclusion analysis uncovers a complex interaction between the gut phageome and the bacteriome

**Fig. 6:**
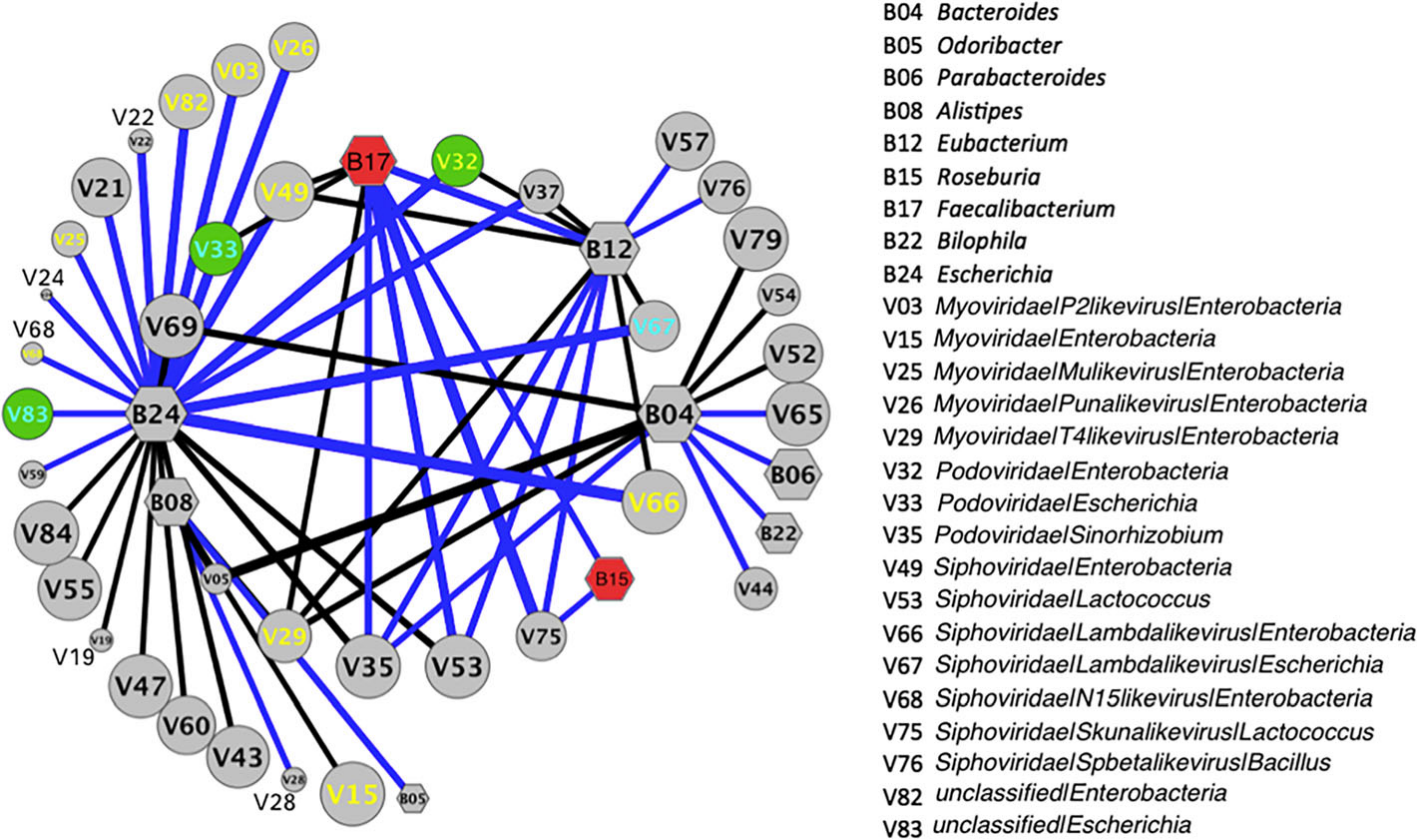
The interaction network of bacterial genera and phage OTUs determined by SparCC. Nodes represent OTUs; round: pOTUs; hexagon: bacterial genera. Only bacterial genera and pOTUs with a high frequency (detected in more than 72.5 samples (50%)) were considered. The co-correlation/exclusion interactions between bacterial genera and pOTUs as well as between bacterial genera were calculated with SparCC. The size of each node is proportional to the number of samples. The width of the edges between nodes is proportional to the correlation values between the nodes, with blue and black indicating the positive and negative correlations, respectively. Only edges corresponding to correlations greater than 0.3 and a *p* value less than 0.01 are shown, and unconnected nodes were omitted. The network layout was calculated by the Cytoscape software using a circular layout. The names of pOTUs with a putative *Enterobacteria* host are marked as yellow, and those with a putative *Escherichia* host are marked as cyan; the nodes representing the bacterial genera enriched in the control group are filled in with red, while the nodes representing the pOTUs enriched in the T2D group are filled in with green. The detailed names of the bacterial genera and only the pOTUs marked with colors were shown on the left panel of the figure. The detailed names of other pOTUs were shown in Additional file 3, Table S6

The predator–prey relationship between phages and bacteria in the gut is central to gut microbiome equilibrium, but the global interactions between phages and bacteria have not yet been well defined. To determine the correlation between the core phages and bacteria (the pOTUs and bacterial genera detected in more than 72.5 samples (50% of 145)), the network between pOTUs and bacterial genera was calculated by SparCC and visualized by Cytoscape [40, 46]. As shown in Fig. 6, in the resulting network of significant (*p* < 0.01) phage–bacteria interactions, each node is either a bacterial genera (hexagon) or pOTU (round). The network has 46 nodes, including 8 bacterial and 38 phage nodes. The genera *Escherichia* (B24) and *Bacteroides* (B04) have the highest numbers of phage interactions. *Escherichia* interacts with 25 pOTUs, including 9 exclusions and 16 co-occurrences. *Bacteroides* has 6 exclusion interactions and 3 co-occurrence interactions. The genus *Faecalibacterium* (B17) has a co-occurrence interaction with the genus *Roseburia* (B15), and these two genera are enriched in the control group (Fig. 5b and Additional file 2: Figure S7). *Faecalibacterium* co-occurs with the pOTUs *Podoviridae|Sinorhizobium* (V35), *Siphoviridae|Lactococcus* (V53), and *Siphoviridae|Skunalikevirus|Lactococcus* (V75), and interestingly, these three pOTUs have co-occurrence interactions with more than two bacterial genera. Of the three pOTUs (*unclassified|Escherichia* (V83), *Podoviridae|Enterobacteria* (V32), and *Podoviridae|Escherichia* (V33)) enriched in the T2D samples (Fig. 5b and Additional file 2: Figure S7), all have co-occurrence interactions with *Escherichia*. In contrast, V33 and V32 have an exclusion interaction with *Faecalibacterium* and *Eubacterium* (B12), respectively. The bacterial hosts of pOTUs with co-occurrence interactions with *Escherichia* are putatively from *Enterobacteria*. *Escherichia* and *Bacteroides* significantly interact with the highest number of pOTUs, suggesting that they are the key microbes dominating the intestine ecosystem.

## Discussion

This study used multiple sophisticated bioinformatic methods to characterize the phageome in fecal samples from T2D and normal Chinese adult individuals. To date, it is the largest study to correlate the gut phageome with T2D, and it substantially expands our understanding of the human gut phageome. More importantly, we demonstrated the significant T2D-specific alterations of the gut phageome. As the primary significance of this study, by large-scale comprehensive bioinformatic analyses, we identified 7 pOTUs associated with T2D patients and illustrated the comprehensive relationships between phages and their bacterial hosts in the gut microbiome.

WCMS data provide more information about phage-bacteria relationships in the gut ecosystem than VLP-derived metagenomic data and thus are suitable for phage-wide association studies. Based on the known phage genomes in the ENADB, we profiled the gut phageome in the WCMS data and VLP-based metagenomic data, resulting in a similar pattern of phageome distribution among the gut samples (Additional file 2: Figure S1). This suggests that the WCMS and VLP data are equally suitable for gut phage detection. However, because of the lack of phage reference genomes and the high complexity of WCMS data, it is challenging to distinguish between the phage and the bacterial sequences in WCMS data. In this study, we used three strategies to overcome the difficulties in recovering large phage scaffolds from the WCMS data. As shown in Fig. 2c, the largest majority of the identified phage scaffolds were assigned taxonomically to the order of *Caudovirales*. Given the important function of terminase in phage DNA packaging of caudoviruses, the gene of terminase can be found on the genomes of various caudoviruses, and thus LST has been widely used as the marker gene for phylogenetic analysis of caudoviruses. To the best of our knowledge, we included the largest number of LSTs extracted from gut samples and all known phage genomes to determine the gut phage phylogeny (Fig. 3 and Additional file 2: Figure S4). This analysis expanded the diversity of gut phages and defined new lineages, implying that the viral studies dependent on the known phages might result in bias of insight into gut phageome.

Most of the gut phages described in this study are novel and are therefore undetected by reference-based methods. Analyses and characterization of these novel phage sequences demonstrated the number of accessory genes carried by the phage scaffolds. This expands our understanding of phage functions in the human gut microbiome. Failure to detect ssDNA phages can be considered as a limitation of the WCMS-based method in the phage detection. We did not intend to distinguish between phage and prophage sequences. Because of the dominance of temperate phages in the human gut ecosystem reported in previous studies, we believe that this study provides more insights into the human gut phageome than VLP-based studies, in terms of detecting both free phages and prophages [12, 14, 47].

Taking advantage of the WCMS data containing valuable information about phages and their hosts, we then paired phage scaffolds with bacterial scaffolds based on the CRISPR-targeting method described by Stern et al. [18]. We first showed in silico a global picture of phage-host specificities in the gut ecosystem via large-scale taxonomic assignment of each pair of scaffolds. As shown in Fig. 4, it can be concluded in silico that most of the gut phages have a narrow bacterial host range within the same genera. A tiny number of phages could cross-infect different bacterial genera (within *Clostridiales*). However, this finding is likely not in conflict with the highly specific nature of phages observed experimentally. The promiscuous phage is capable of evolving rapidly to infect new hosts by recruiting or mutating a new tail fiber gene.

Interactions between bacterial phages and their hosts dominate the gut ecosystem. Therefore, it is critical to identify the relationships between phages and bacteria to understand the potential role of phages in the development of T2D. However, only some of the phage scaffolds could be paired with the bacterial scaffolds based on the CRISPR-targeting method. Moreover, by mapping the reads across 145 samples to the identified phage scaffolds, we observed that most of the identified phage scaffolds exhibited high breadth of coverage and depth of coverage only in the samples where they were detected (Additional file 2: Figure S4a and b), suggesting that the gut phageomes are highly individualized, as described previously [10, 12]. Although we observed a significant increase in the number of phages in T2D samples based on the identified phage scaffolds (Fig. 5c), alterations of the phageome and bacteriome could not be explained by some particular phage taxa. Thus, we tentatively defined pOTUs to determine the association between phages and bacteria. We identified 7 pOTUs that were significantly increased in the T2D group. Additionally, we estimated the richness of the gut phageome in T2D and control samples but did not find significant differences in the richness of the gut phageome between the two groups (Additional file 2: Figure S8). Therefore, given that temperate phages are dominant in the gut ecosystem [10, 13], we hypothesize that T2D-related factors enriched in the gut of T2D patients cause lysogenic phages to switch to the lytic cycle. Further investigations are required to determine the possible mechanisms underlying.

It is of interest to note that we found that the gut phages’ pattern with regard to T2D is not mirrored in bacteria. According to the hypothesis of “Kill the Winner,” phages are well known to be main predators of gut bacteria [48]. However, many recent studies suggested that bacteriophages play roles in intestinal physiology that are far more important than the alteration of bacterial communities by their infection. Norman et al. reported inflammatory bowel disease-specific alterations in the enteric virome [14]. Phages residing in mucosal surfaces can provide non-host-derived immunity against bacterial infections [26]. A growing number of evidences showed that phages are able to directly affect human [14, 19, 20, 25–27]. For example, phages can act as antigens to stimulate host immunity and inflammation [49]. Here, we also found that the gut bacterial phages carried accessory genes responsible for human pathogenesis or human fitness (Additional file 2: Figure S6, Additional file 3: Table S3). In the light of this, it is not necessary to be surprising that the patterns of the phageome and bacteriome were distinct from each other (Fig. 5), and that extensive co-occurences/exclusions between phages and bacteria existed in gut ecosystem (Fig. 6). Gut microbiota is a great complex community, in which, the commensal, mutualistic, and symbiotic relationships between different bacterial taxa, phages and human, phages and bacteria, bacteria and human, etc., might be established. Particularly, the genus of *Bacteroides* is the most abundant bacteria in the gut, and thus interacts with other members extensively. Figure 6 shows that many phages are associated with the genus of *Bacteroides*, likely reflecting indirect associations between *Bacteriodes* and other phages. Thus, our study implies a new direction to revisit the T2D risk. And we may have to take virome’s impacts on human gut microbiome into consideration when we attempt to develop therapeutic approaches against T2D via manipulating gut microbiome.

## Conclusions

Here, for the first time, we correlated the gut phageome with T2D. Instead of a reciprocal relationship of phage specific to its bacterial host, a complex core interaction among bacteria and phages was revealed by a co-occurrence/exclusion analysis of bacterial genera and pOTUs. The T2D-associated changes in the phageome cannot simply be explained as co-variation with their altered bacterial hosts. These results imply that in addition to the cardinal and highly specific bacteria–phage interaction, other mechanism(s) may govern the phageome in the gut ecosystem. This study suggests that we should pay more attention to the role of phages in human microbiome studies and indicates the potential of gut phages in diagnostic and therapeutic applications.

## Additional files

Additional file 1: This pdf file contains supplementary Material and Methods for the detailed descriptions of identification of the large phage scaffolds and definition of the pOTUs. (DOCX 101 kb)

Additional file 2: This pdf file contains the following supplementary figures: S1-S8. Legends for these figures are presented at the beginning of Additional file 1. (DOCX 3421 kb)

Additional file 3: This xlsx file contains the following supplementary spreadsheets, each included as a separate tab in a single Microsoft Excel file: TableS2-TableS5. (XLSX 242 kb)

## Abbreviations

BGR: Bacterial genus ratio
BPGDB: Bacterial and Phage Gene Database
ENA: European Nucleotide Archives
ENADB: European Nucleotide Archives genome database
ENAPDB: European Nucleotide Archives protein database
EPSGDB: Expanded Phage-Specific Gene database
IBD: Inflammatory bowel disease
LST: Large subunit terminase
NGS: Next-generation sequencing
PFR: Phage family ratio
pOTU: Phage operational taxonomic unit
SDB: Spacer DataBase (SDB)
T2D: Type 2 diabetes
VLP: Virus-like particles
WCMS: Whole-community metagenomic sequencing
