# Supplementary material for: A human gut phage catalog correlates the gut phageome with type 2 diabetes
Source: Microbiome. 2018 Feb 1;6:24. doi: 10.1186/s40168-018-0410-y (PMC5796561; doi:10.1186/s40168-018-0410-y)
Supplement: Supplementary file 1 — This pdf file contains supplementary Material and Methods for the detailed descriptions of identification of the large phage scaffolds and definition of the pOTUs. (DOCX 101 kb) [file 40168_2018_410_MOESM1_ESM.docx]

**Abbreviations:**

T2D Type 2 Diabetes

pOTU Phage Operational Taxonomic Unit

bOTU Bacterial Operational Taxonomic Unit

ENA European Nucleotide Archives

ENADB European Nucleotide Archives genome database

ENAPDB European Nucleotide Archives protein database

VLP Virus-like Particles

NGS Next generation sequencing

WCMS Whole community metagenomic sequencing

IBD Inflammatory bowel disease

PFR Phage family ratio

BGR Bacterial genus ratio

EPSGDB Expanded Phage-Specific Gene database

LST Large subunit terminase

BPGDB Bacterial and Phage Gene DataBase

SDB Spacer DataBase (SDB)

**Material and Methods**

**Identification of the large phage scaffolds**

To distinguish the sequences with phage origin from WCMS datasets is challenging. We developed three strategies to identify large phage scaffolds from the assembled scaffolds of the T2D study (gigadb.org/dabaset/100036)(Figure 1.).

**Strategy I**: The sequences of CRISPR were searched in Scaffold datasets by CRISPR recognition tool locally [[1](#_ENREF_1)]. All the spacer sequences (42479) which probably originated from phage or plasmid sequences were collected from the 145 WCMS samples, averagely 292(±220) from 10 to 1038 per sample, and then deduplication by software CD-HIT resulted in finding 36933 spacers[[2](#_ENREF_2)]. All the reads were queried against the spacer sequences. The reads with hits to at least one spacer sequence were extracted and then formatted as a Spacer DataBase (SDB). All the assembled larger scaffolds (>=10k) then were compared against the SDB for phage scaffold searching. After filtering procedure to remove those with CRISSPR assays, the remaining scaffolds with hits in SDB were selected as phage sequence candidates for downstream analysis.

**Strategy II**: All Virus-like Particle (VLP) metagenomic reads were mapped to the large scaffolds (>=10 kb). The scaffolds with high breadth-of-coverage (>=40%) by the reads from VLP metagenomes were selected as phage sequence candidates.

**Strategy III**: All gene sequences encoded by the larger scaffolds (>=10K) were compared against the genes encoded by all known phage genomes downloaded from ENA with cutoff 1e-10 by the software Diamond[[3](#_ENREF_3)]. The scaffolds with at least one gene hit to phage genes were selected as phage scaffold candidates. All the scaffolds selected as phage candidates in above steps were combined as large phage candidates I and then were dedupulicated for further phage scaffold identification.

**Construction of Bacterial and phage gene database (BPGDB):** All the genes predicted from all known bacterial genomes (8719483 genes, from 2642 bacterial genomes) downloaded from NCBI (<ftp://ftp.ncbi.nlm.nih.gov/genomes/Bacteria.(07-10-2015>)), were compared against (cutoff 1e-10) all phage and prophage gene sequences that were downloaded from EBI (228064, from 2469 phage genomes) and PHAST, respectively[[4](#_ENREF_4)]. The bacterial genes (1298259) with hits to the phage genes were then removed. The remaining bacterial genes (marked as B) and the phage genes (marked as P) were further formatted as Bacterial and Phage Gene DataBase (BPGDB).

The genes encoded by the phage scaffold candidates I were queried against the BPGDB with cutoff 1e-10. Each scaffold was calculated bacterial index that was defined as the ratio of the number of genes with best hits to bacterial (B) genes among the number of all genes encoded by the scaffold. A scaffold with high bacterial index (>=0.7) will be assigned as bacterial sequences and will be excluded for downstream analysis. The remaining scaffolds (n=2796) with bacterial index less than 0.3 were selected as phage scaffold candidates II.

The phage scaffold candidates II were clustered, and the scaffolds with 80% breadth-of-coverage and 80% identities belong to one group and the longest scaffold was selected as representative for this group for downstream analysis. The genes encoded by the phage scaffold candidates II were queried against GenBank nr database with cutoff 1e-05. The genes of phage scaffolds got multiple hits in GenBank database and each scaffold were manually checked. Accordingly, the scaffolds encoding genes with functions for plasmids were assigned as plasmid scaffolds and then were excluded for downstream analysis (n=59).

The remaining scaffolds were assigned as phage scaffolds candidates III for downstream analysis, resulting in 2567 phage scaffolds. 171 scaffolds were discarded due to ambiguous annotation in GenBank nr database.

**Definition of the Phage Operational Taxonomic Units (pOTUs)**

It is unlikely to determine the global connections between phages and their hosts in gut metagenomes due to the culture-independent nature of metagenomic analysis. Only a fragment of phage-host specificities can be determined by CRISPR-targeting method.

Therefor, to determine the association between phages and bacteria, we tentatively defined pOTUs which refer to a group of phages sharing with the same taxonomic names at all five levels (group|order|family|subfamily|genus) and hosts within same genus. The updated Phage orthologous groups (POGs) contain 57439 genes that can be grouped into 4542 clusters of orthologous genes from 1028 bacterial and archaea phage genomes, among of which, more than 60% of the genes (35415) with high frequency of being found in phage genomes can be assigned as phage-specific genes[[5](#_ENREF_5)]. The latest European Nucleotide Archive (ENA) (5-May-2015) contains 2501 whole phage genomes. We expanded the phage-specific genes by comparing the genes encoded by the 2501 phage genomes of ENA against virus-specific genes in POGs via psiblast with hit e-values less than 1e-10 and all the genes with hits were formatted as Expanded Phage-Specific Gene database EPSGDB. According to the phage classification defined by ICTV, phages can be taxonomically assigned at six level, i.e. , group, order, family, subfamily, genus and species. Each of 2501 phage genomes downloaded from ENA were assigned at all taxonomic levels of group, order, family, subfamily and genus. If the name of one level is unknown or unidentified, the taxonomic name will be given as unclassified plus level name. The pOTU was defined as the collective including all phages with same taxonomic name at all five levels (group|oder|family|subfamily|genus) and sharing same genus host. Thus, the pOTU names were given as all five level names plus genus name of bacterial hosts. For example, Lactococcus phage bIL170 (GenBank accession No. AF009630) was isolated from a bacterium of Lactococcus. Accordingly, the pOUT of this phage can be assigned as: dsDNAviruses|Caudovirales|Siphoviridae| subfamily_unclassified|Skunalikevirus|Lactococcus. All the metagenome reads were translated and compared against EPSGDB by the software DIAMOND with flags as e-value<=1e-05 and identity>60%[[3](#_ENREF_3)].

To estimate the ratio of phage/bacterial number is important to characterize the correlation between preys and predators. The total bacterial number in a metagenome was estimated by searching 16S rRNA gene reads by BLASTn (cutoff 98% length alignment, 90% identity) against Greengene database.

The relative number of EPSGDB gene (RNGi) was estimated by

*RNGi=(Ni/Li)/Np,*

Ni, the read number of phage specific gene *i,*

*Li,* the length of gene *i, Np,* the number 16s rRNA gene reads.

The relative number of a phage (aRNGPj) was equal to that of its encoding gene, and if the genome encodes multiple EPSGDB genes that were hit by reads, then the highest relative number of gene was used.

*a*RNGPj= Maximum(RNGi)

*aRNGPj, the relative number of phage j*

The relative number of pOTU (aOTUn) was calculated by summing all the relative numbers of all phage genomes belonging to the pOTU. The relative number of a phage family was calculated by summing all the relative number of all pOTUs belonging to the phage family.

*a*OTUn= Σ_j_(aRNGP1+……aRNGPj)

*a*FAMILYm= Σ_n_ (pOTU1+……pOTUn)

*aOTUn, the relative number of phage OTU n.*

*aFAMILYm, the relative number of phage family m.*

To assess the phage richness in 145 samples, a rarefaction curve was generated by the R package Vegan based on the Chao2 richness estimator.

Table S1. Metagenomic datasets used in this study

| **Data** | **Link** | **Data summary and roles in this study** | **Ref** |
| --- | --- | --- | --- |
| NCBI SRA | http://www.ncbi.nlm.nih.gov/sra?term=SRA045646 | Whole community metagenomic datasets generated from the samples of patients with type II Diabets and normal controls. | [[6](#_ENREF_6)] |
| GigaDB | http://gigadb.org/dataset/100036 | Scaffolds assembled from the metagenomic datasets. | [[6](#_ENREF_6)] |
| NCBI SRA | http://trace.ncbi.nlm.nih.gov/Traces/sra/?study=SRP021107 | Virus-like particle metagenomic datasets | [[7](#_ENREF_7)] |
| EMBL-EBI | http://www.ebi.ac.uk/ena/data/view/PRJEB7772 | Virus-like particle metagenomic datasets | [[8](#_ENREF_8)] |
| NCBI SRA | http://www.ncbi.nlm.nih.gov/sra/?term=SRX020505, SRX020504 | Virus-like particle metagenomic datasets | [[9](#_ENREF_9)] |
| NCBI Viral genome | http://www.ncbi.nlm.nih.gov/genome/viruses/ | All genomes of known viruses |  |
| NCBI COG | http://www.ncbi.nlm.nih.gov/COG/ | Clusters of orthologous groups | [[10](#_ENREF_10)] |
| NCBI POG | http://www.ncbi.nlm.nih.gov/COG/ | Phage orthologous groups | [[5](#_ENREF_5)] |
| EBI | http://www.ebi.ac.uk/genomes/phage.html | All genomes of known bacterial phages |  |
| EMBL-EBI | http://pfam.xfam.org/ | Pfam database, functional annotation | [[11](#_ENREF_11)] |

Reference:

1. Bland, C., et al., *CRISPR recognition tool (CRT): a tool for automatic detection of clustered regularly interspaced palindromic repeats.* BMC Bioinformatics, 2007. **8**: p. 209.

2. Li, W. and A. Godzik, *Cd-hit: a fast program for clustering and comparing large sets of protein or nucleotide sequences.* Bioinformatics, 2006. **22**(13): p. 1658-9.

3. Buchfink, B., C. Xie, and D.H. Huson, *Fast and sensitive protein alignment using DIAMOND.* Nat Methods, 2015. **12**(1): p. 59-60.

4. Zhou, Y., et al., *PHAST: a fast phage search tool.* Nucleic Acids Res, 2011. **39**(Web Server issue): p. W347-52.

5. Kristensen, D.M., et al., *Orthologous gene clusters and taxon signature genes for viruses of prokaryotes.* J Bacteriol, 2013. **195**(5): p. 941-50.

6. Qin, J., et al., *A metagenome-wide association study of gut microbiota in type 2 diabetes.* Nature, 2012. **490**(7418): p. 55-60.

7. Minot, S., et al., *Rapid evolution of the human gut virome.* Proc Natl Acad Sci U S A, 2013. **110**(30): p. 12450-5.

8. Norman, J.M., et al., *Disease-specific alterations in the enteric virome in inflammatory bowel disease.* Cell, 2015. **160**(3): p. 447-60.

9. Minot, S., et al., *The human gut virome: inter-individual variation and dynamic response to diet.* Genome Res, 2011. **21**(10): p. 1616-25.

10. Galperin, M.Y., et al., *Expanded microbial genome coverage and improved protein family annotation in the COG database.* Nucleic Acids Res, 2015. **43**(Database issue): p. D261-9.

11. Finn, R.D., et al., *Pfam: the protein families database.* Nucleic Acids Res, 2014. **42**(Database issue): p. D222-30.
