# Supplementary material for: A human gut phage catalog correlates the gut phageome with type 2 diabetes
Source: Microbiome. 2018 Feb 1;6:24. doi: 10.1186/s40168-018-0410-y (PMC5796561; doi:10.1186/s40168-018-0410-y)

**Figure Legends**

**Fig S1.** Breadth of coverage of ENA phage genomes by the WCMS reads. Breadth-of-coverage: the percentage of phage genome was covered by the mapped reads. Only the phage genomes with high prevalence (with hits by at least one read from more than 10 % of all samples) among samples were shown. Dark arrow represents the VLP dataset; N, control samples; Y, T2D samples. Each row represents one phage genome and each column represents one sample. ssDNA phages are not shown. A: Family_Myoviridae; B: Family_Podoviridae; C: Family_Siphoviridae; D: Genus_P2likevirus; E: Genus_Punalikevirus; F: Genus_Lambdalikevirus; G: Unclassified.

**Fig S2.** The relative abundances of phage families in gut samples based on pOTU assignment.

We defined pOTUs by an Expanded Phage-Specific Gene database (EPSGdb) according to the Phage Orthologous Groups (POGs). The pOTU was defined as the collection including all phages with same taxonomic name at all five levels (group|oder|family|subfamily|genus) and sharing same genus host. The relative number of pOTU was calculated by summing all the relative numbers of all phage genomes belonging to the pOTU and then were normalized by the number of 16s rRNA gene reads in the sample.

**Fig S3.** Large phage scaffolds unique to individuals shown by breadth-of-coverage (a) and depth of coverage (b).

a. The breadth of coverage of each phage scaffold, showing the percentage of the phage scaffold can be mapped by the reads in one samples; b. The depth of coverage of each phage scaffold, showing the number of reads was mapped to phage scaffold in one sample.

**Fig S4.** Phylogenetic trees of gut phages based on large subunit terminases.

**(A)** Terminase_6; **(B)** Terminase_gpa; Uncultured, black; Myo, green; Siph, red; Pod, blue. Unclassified cultured, yellow. Maximum-likelihood trees of all identified large subunit terminases from the putative gut phage scaffolds and ENA phage genomes. The branches in the tree colored as red denote the sequences from ENA phage genomes. The trees were built by the software MEGA and the viewed by Figtree. Bootstrap was set up as 1000.

**Fig S5.** Genes on phage scaffolds annotated based on COG database.

J, translation, including ribosome structure and biogenesis; L, replication, recombination and repair; K, transcription; O, molecular chaperones and related functions; M, cell wall structure and biogenesis and outer membrane; N, secretion, motility and chemotaxis; T, signal transduction; P, inorganic ion transport and metabolism; C, energy production and conversion; G, carbohydrate metabolism and transport; E, amino acid metabolism and transport; F, nucleotide metabolism and transport; H, coenzyme metabolism; I, lipid metabolism; D, cell division and chromosome partitioning; R, general functional prediction only; S, no functional prediction.

**Fig S6.** The genes encoded by the sequences of phages specific to *Bacteroides spp.* in gut.

The red color represents the genes related to recombination and replication of DNA; the pink and dark-green colors represent the marker genes of the phages, respectively; the light-green color represents the genes related to methylase; the light-blue color represents the “gp” genes; the orange color represents mostly enzymes; the dark color represented the genes of hypothetical proteins; the purple color represents the genes of transposase.

**Fig S7.** Bacterial taxa differentially enriched between T2D and controls computed by LEfSe.

LDA scores represent the degree of consistent difference in relative abundance between taxa in the two groups of analyzed microbial communities. The histogram identifies which clades among all those detected as statistically and biologically differential explain the greatest differences between communities. The software LEfSe (LDA score>3) detected that clostridial bacteria including *Roseburia intestinalis*, *R. inulinivorans*, *Faecalibacterium prausnitzii*, as well as non-clostridial bacteria - *Haemophilus parainfluenzae*, and *Bacteroides eggerthii* were enriched in control sample*s*; while actinobacteria *Bifidobacterium breve* and *Gordonibacter pamelaeae*, *Lactobacillus amylovorus*, *Enterococcus faecalis*, *Eubacterium limosum*, *L. delbrueckii*, *Acidaminococcus fermentans*, *Akkermansia muciniphila*, *Coprobacillus bacterium*, *Anaerofustic stercorihominis*, *Pyramidobacter piscolens* were enriched in T2D samples.

**Fig S8.** Rarefaction curves of the phageome in the T2D (Y) and control (N) groups.

Based on (a) the number of ENA genomes with high breadth-of-coverage in the metagenomes; (b) the number of defined pOTUs in the metagenomes; (c) based on the number of the identified large phage Scaffolds. The rarefaction cures were generated by the R package Vegan based on the Chao2 richness estimator.

To determine the differences of phage profiles between the phenotypes normal samples and diabetes samples, two rarefaction curves were generated. There may be a relatively limited pool of common phages present in many individuals along with a much larger set of rare phages that will require significantly more sequencing to reach saturation.

Fig S1.


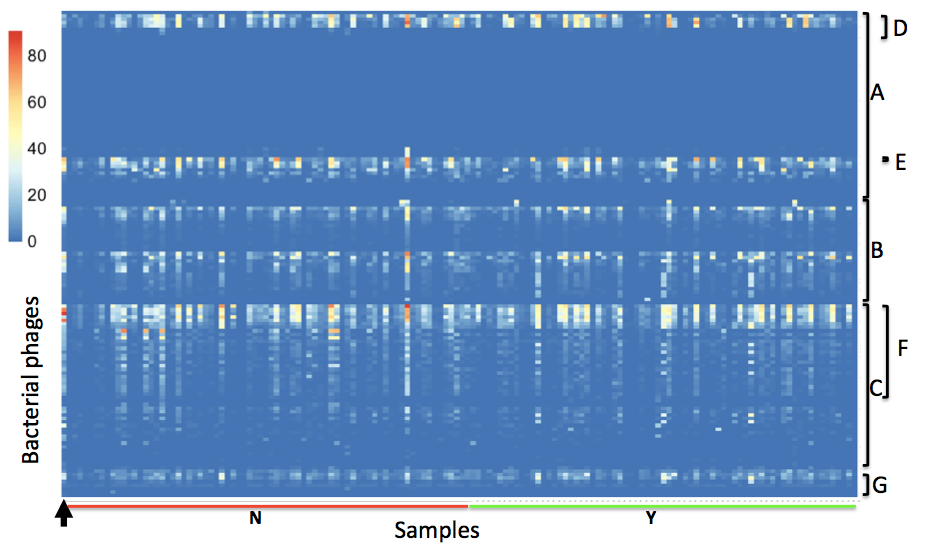


Fig S2.


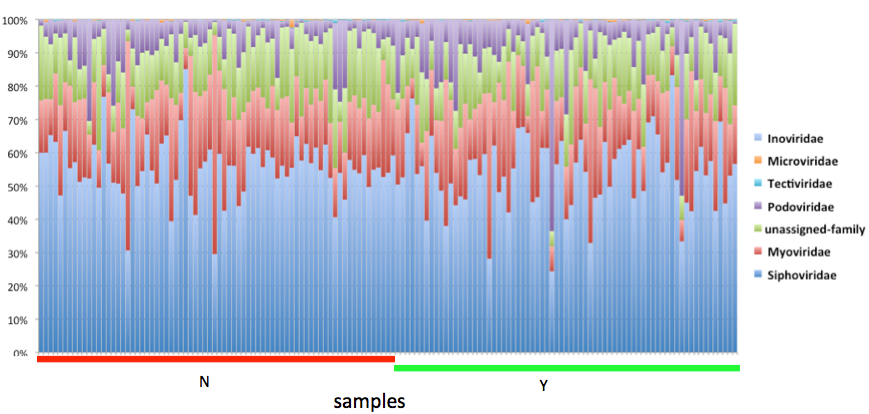


Relative abundance

Fig S3.


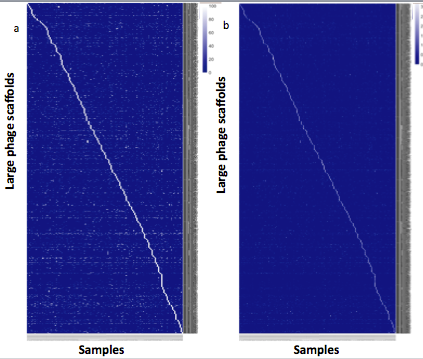


Fig S4.


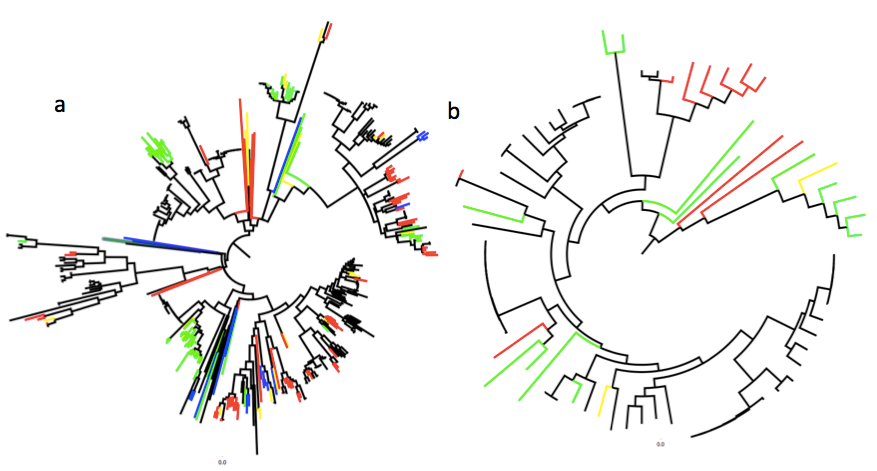


Fig S5.


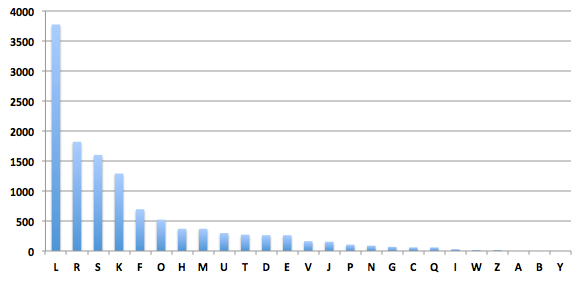


Fig S6.


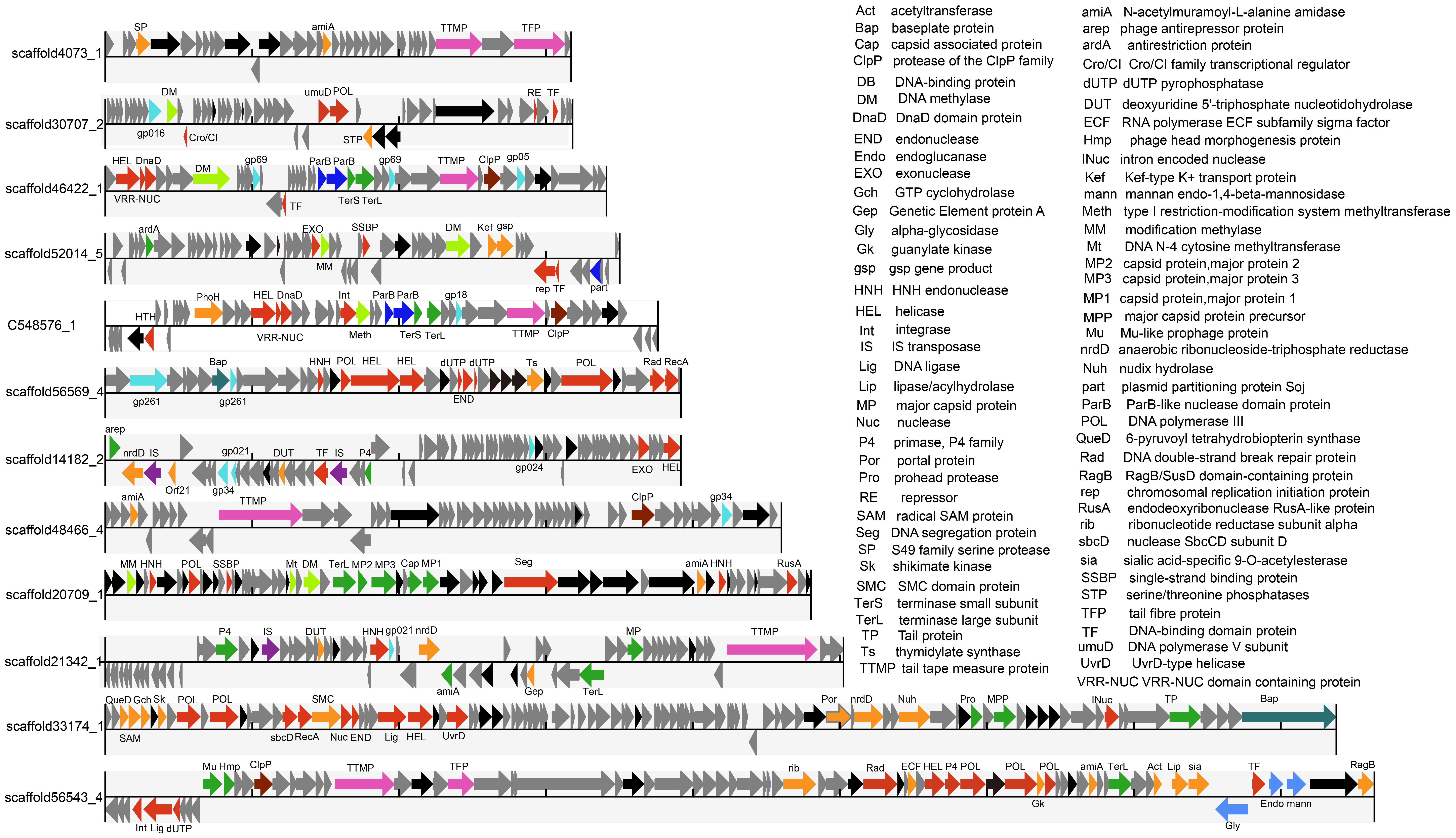


Fig S7.


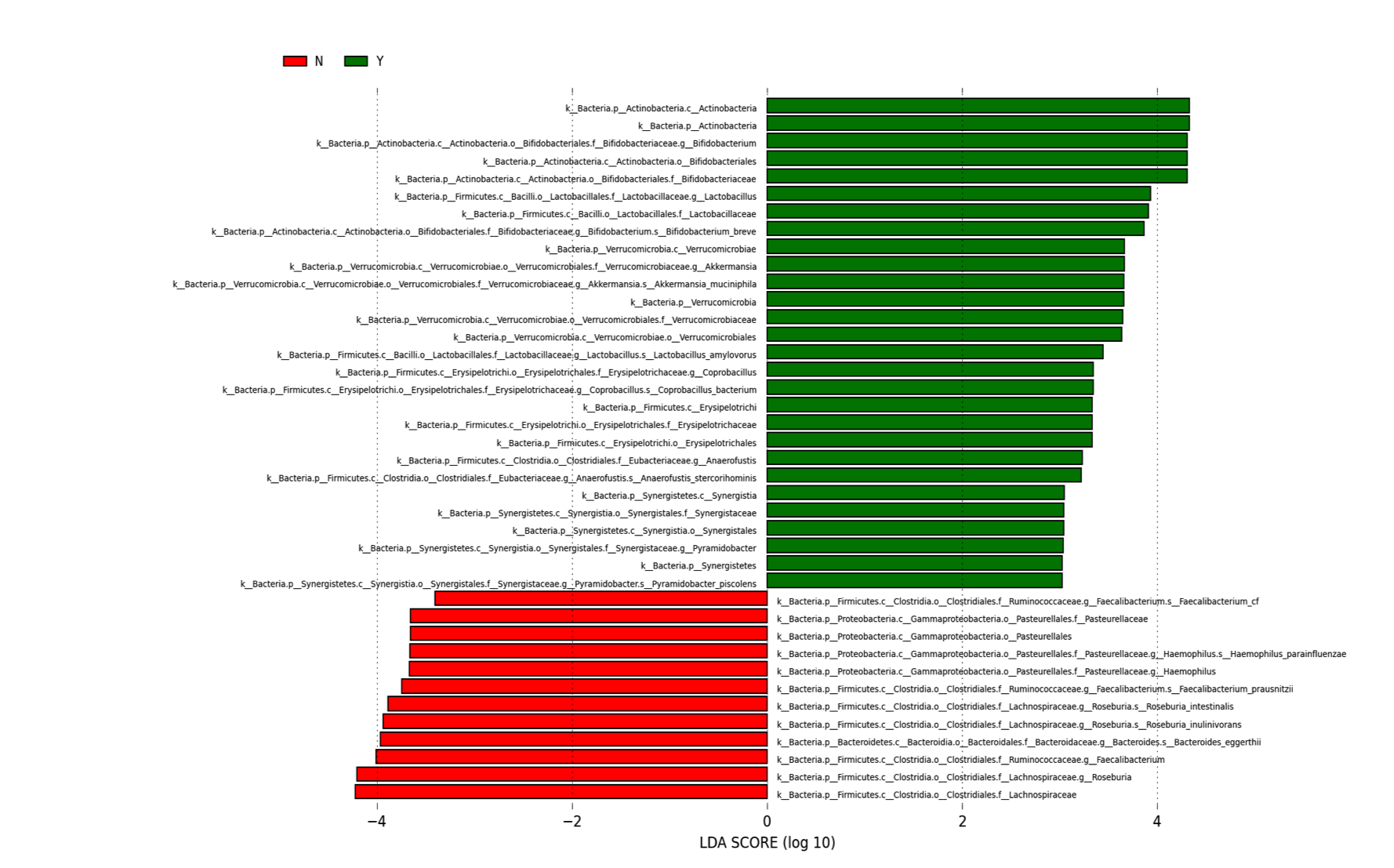


Fig S8.


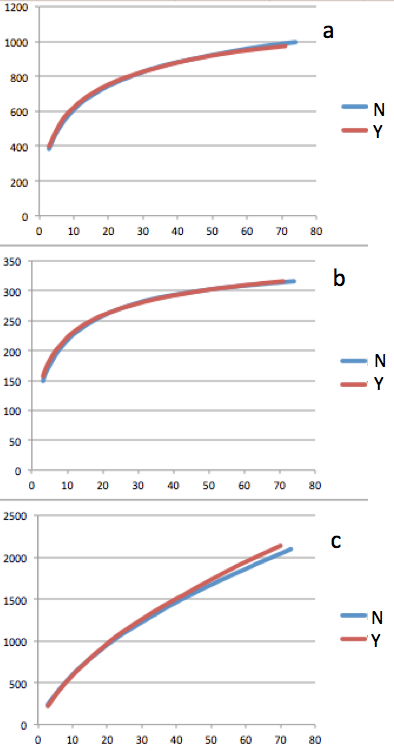

Supplement: Supplementary file 2 — This pdf file contains the following supplementary figures: S1-S8. Legends for these figures are presented at the beginning of Additional file 1. (DOCX 3421 kb) [file 40168_2018_410_MOESM2_ESM.docx]
